# “Together against Tuberculosis”: Cascade of Care of Patients Referred by the Private Health Care Providers in the Kyrgyz Republic

**DOI:** 10.3390/tropicalmed8060316

**Published:** 2023-06-10

**Authors:** Dinara Madybaeva, Aiymgul Duishekeeva, Anna Meteliuk, Aizat Kulzhabaeva, Abdullaat Kadyrov, Natalia Shumskaia, Ajay M. V. Kumar

**Affiliations:** 1 Public Foundation “AFEW KG”, 14 JalArtis, 103, Bishkek 720047, Kyrgyzstan; natalya.shumskaya@afew.kg; 2 Public Foundation “KNCV KG”, 121 Dzhantoshev St., Bishkek 720020, Kyrgyzstan; a.duishekeeva@kgma.kg (A.D.); a.kuljabaeva@kgma.kg (A.K.); 3 Department of Phthisiology, Kyrgyz State Medical Academy, 92 Akhunbaev St., Bishkek 720020, Kyrgyzstan; 4 International Charitable Foundation Alliance for Public Health, 03150 Kyiv, Ukraine; meteliuk@aph.org.ua; 5 Department of Public Health, Kyrgyz State Medical Academy, 92 Akhunbaev St., Bishkek 720020, Kyrgyzstan; 6 National Center for Phthisiology of MoH Kyrgyzstan, Ahunbaev St., 90a, Bishkek 720064, Kyrgyzstan; tbkgprogram@gmail.com; 7 International Union Against Tuberculosis and Lung Disease, 2 Rue Jean Lantier, 75001 Paris, France; akumar@theunion.org; 8 International Union Against Tuberculosis and Lung Disease, South-East Asia Office, C-6 Qutub Institutional Area, New Delhi 110016, India; 9 Department of Community Medicine, Yenepoya Medical College, Yenepoya (Deemed to be University), University Road, Deralakatte, Karnataka, Mangalore 575018, India

**Keywords:** Kyrgyz Republic, TB presumptive, failure, operational research, SORT IT, key population, private sector

## Abstract

Until 2021, in the Kyrgyz Republic, tuberculosis (TB) was diagnosed and treated only in the public sector. With funding support of the STOP–TB partnership, the private providers in four regions of the country and Bishkek city were mapped, trained and incentivized to screen for and identify presumptive TB patients and refer them to the public facilities for diagnosis and treatment. In this study, we describe the cascade of care of such patients. This was a cohort study involving secondary analysis of routine data. Of 79,352 patients screened during February 2021–March 2022, 2511 (3%) had presumptive TB, of whom 903 (36%) were not tested for TB [pre-diagnostic loss to follow-up]. A total of 323 (13%) patients were diagnosed with TB, of whom, 42 (13%) were not started on treatment [pre-treatment loss to follow-up]. Among 257 patients eligible for outcome assessment, 197 (77%) had treatment success, 29 (11%) were lost-to-follow-up, 13 (5%) died, 4 (2%) had treatment failure and 14 (5%) were not evaluated. While this donor-funded, pioneering initiative was successful in engaging the private sector, we recommend that the national TB programme scales up the initiative nationally with dedicated budgets, activities and plans to monitor progress. Qualitative research is urgently needed to understand the reasons for the gaps in the care cascade.

## 1. Introduction

Tuberculosis (TB) continues to remain a global public health problem. It is the second most common cause of death worldwide among infectious diseases, next only to COVID-19. According to the World Health Organization’s (WHO) Global Tuberculosis Report, an estimated 10.6 million people fell ill with TB worldwide in the year 2021 (an increase of 4.5% compared to 2020) and 1.6 million people died due to TB [1]. This is the first time in many years that the estimated incidence of TB has shown an increase, primarily due to COVID-19-related disruptions to health systems and services [1]. For the same reason, the number of TB patients diagnosed and notified has decreased. This suggests that there is an increase in the number of tuberculosis cases which are undiagnosed and untreated, and as per the Global TB report, this accounted for 40% of all incident cases in 2021 [1]. This has two important consequences: (i) increased deaths among undiagnosed/untreated cases and (ii) continued transmission of infection in the community, which may lead to increased numbers of active TB disease in the future.

The situation in Kyrgyzstan, a lower middle-income country in Central Asia and considered a high priority TB country in the European region, is no different. Of the 8500 estimated incident cases of TB in 2021, only 4500 were detected and notified, indicating that about 46% of the incident cases were undiagnosed and untreated [1]. One of the reasons for this gap is the suboptimal engagement of private health care providers in the management of TB. Until recently, TB was diagnosed and treated only in the public sector. This changed on 31st December 2020, with an order of the Ministry of Health which permits the private health care providers to identify and refer presumptive tuberculosis patients to the public health facilities for TB diagnosis and treatment.

In the year 2021, a project titled “Together Against TB” was launched and a model of engagement of private providers was implemented, with funding support from the STOP TB Partnership. Under this project, private-for-profit health care providers in four regions of Kyrgyzstan and Bishkek city were mapped, trained and incentivized to screen and identify presumptive TB patients as per the diagnostic algorithm of the national TB programme, and refer them to the public health facilities for diagnosis and treatment.

This project provides an excellent opportunity to assess the cascade of care and quantify the gaps at each stage of the TB care cascade. Evidence from other countries indicates two major gaps: pre-diagnostic loss to follow-up (LFU), which indicates the gap between identification as ‘presumptive TB’ and getting tested for TB, and pre-treatment LFU, which indicates the gap between diagnosis and treatment [2]. While there are many studies reporting on pre-treatment loss to follow-up, studies on pre-diagnostic loss to follow-up are limited [3]. Evidence from the private health sector is scarce [4].

There is no previous published study on TB care cascade from Kyrgyzstan. Such evidence is useful for two reasons: (i) to identify gaps in the care cascade and take steps for improving the quality of care of TB patients; (ii) to optimize the implementation model of private-provider engagement and take decisions on nationwide scale-up. Hence, we decided to undertake this study. The aim of the study was to describe and quantify the gaps in the cascade of care among presumptive TB patients identified and referred by the private health care providers in Kyrgyzstan. Specific objectives were to determine, among patients attending the private health facilities in four selected regions and Bishkek city of Kyrgyzstan, between February 2021 and March 2022: (i) the number of individuals screened for TB and, among them, the number (proportion) identified as presumptive TB; (ii) among presumptive TB patients, the number (proportion) of individuals tested for TB using sputum microscopy and/or Xpert MTB/RIF and the number (proportion) diagnosed as TB; (iii) among diagnosed TB patients, the number (proportion) started on treatment; (iv) treatment outcomes; and (v) the demographic and clinical factors associated with pre-treatment loss to follow-up and unsuccessful treatment outcomes.

## 2. Materials and Methods

### 2.1. Study Design

This was a cohort study involving analysis of secondary data collected by the project.

### 2.2. Study Setting

The Kyrgyz Republic is a land-locked, lower-middle-income country in Central Asia and has a population of 6.7 million [5]. The country is divided into seven oblasts or regions (Chuy, Osh, Issyk-Kul, Naryn, Talas, Jalal-Abat, Batken) and two cities (Bishkek and Osh). The Gross Domestic Product per capita in Kyrgyzstan was 1276 US dollars in 2021 [6]. The terrain of Kyrgyzstan is dominated by the Tian Shan and Pamir mountains, which together occupy about 65% of national territory.

The private health sector is largely concentrated in urban areas, especially the two largest cities: Bishkek (~1 million population) and Osh (~250,000 population). Private health insurance is generally not prevalent in the country and individuals have to pay out-of-pocket in order to access health care services from private health care providers.

### 2.3. Specific Setting

**Figure 1:**
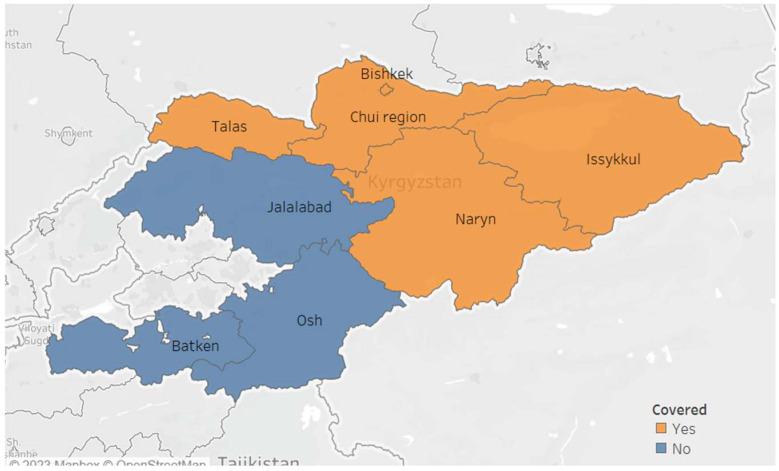
Map of the Kyrgyz Republic showing the regions covered by the “Together against TB” project engaging private providers in tuberculosis in 2021–2022.

The “Together against TB’ project was implemented in four oblasts (Chuy, Issyk-Kul, Naryn, Talas) and the city of Bishkek (Figure 1). These regions were selected based on convenience and feasibility of implementing the project. The project was led by AFEW (AIDS Foundation East-West, the principal recipient of the grant) in collaboration with KNCV-KG Tuberculosis Foundation. Before the launch of the project, the project team underwent training conducted by STOP TB Partnership (the donor) on all the specifics of the project, including an overview of the national TB program and the requirements of recording, reporting, monitoring and evaluation of the project. Within the project area, there were a total of 89 private health facilities, of which 55 (62%) were engaged in the project. A total of 83 providers (which included general physicians, pulmonologists and nurses) underwent dedicated training for two days on national TB program guidelines and project activities. Providers who could not attend the training were visited by the project staff and sensitized on-the-job.

#### 2.3.1. Screening and Identification of Presumptive TB Patients

It is important to screen all patients at outpatient departments who passively report any cough, irrespective of duration, in order to increase TB case finding and reduce TB transmission and mortality [7,8]. The main task of private providers within the project was to screen for symptoms to identify TB presumptive cases. A working group of the National Phthisiology Center developed guidelines for private medical providers on the algorithm for detecting and diagnosing TB.

As per the guidelines, all patients who visited the private health facilities and consulted the general physician or pulmonologist of the clinic were screened for symptoms suggestive of TB (cough of ≥2 weeks, fever, weight loss, night sweats, chest pain), regardless of the reason for their visit. If a patient had persistent cough for more than 2 weeks and his/her condition did not improve after a course of broad spectrum antibiotics, people were investigated using: (i) Xpert MTB/RIF assay, (ii) microscopy and (iii) chest X-ray.

People with symptoms other than a cough were examined by a clinician and, based on detailed medical history (such as contact history with a case of TB) and physical examination findings, a decision was made of whether the patient was a ‘presumptive TB’ or not. All patients considered ‘presumptive TB’ were requested to undergo chest radiography, if not already performed. In addition, patients undergoing chest radiography for any reason and found to have shadows suggestive of TB were also considered ‘presumptive TB’ and investigated further.

#### 2.3.2. Sputum Collection and Transport

Presumptive TB patients were instructed to collect two sputum specimens (collected both early morning the next day) in sputum containers and submit it either to the private health facility or the public laboratory, as per the convenience of the patient. Sputum specimens submitted to the private health facility were transported by the nurse (human carrier) or couriered to the public laboratory for testing. Of the two specimens, one was tested using sputum microscopy and another was tested using Xpert MTB/RIF test. There were 10 GeneXpert laboratories and 47 microscopy laboratories in the project areas.

#### 2.3.3. Diagnosis and Treatment of TB

All patients who were positive for acid-fast bacilli on sputum microscopy or positive for TB by Xpert MTB/RIF were considered as ‘bacteriologically confirmed’ tuberculosis cases. People who were not positive on microscopy or Xpert, but had shadows suggestive of TB on chest radiography and persistent symptoms after an unsuccessful course of broad spectrum antibiotics, were considered ‘clinically diagnosed’ tuberculosis cases. The decision of clinical diagnosis was made by a committee of TB doctors. People with symptoms and signs suggestive of extra pulmonary TB were investigated further and a diagnosis was made based on the results of investigations and clinical judgement. Patients found to have rifampicin resistance on Xpert MTB/RIF test were investigated further (using line probe assay and liquid culture and phenotypic drug susceptibility tests) for presence of resistance to other first-line and second-line drugs. These tests were available only at the reference laboratory at Bishkek city. This required collection of fresh sputum samples and transportation to the reference laboratory. Based on the results, the type of resistant TB (isoniazid mono resistant TB, poly resistant TB, multidrug-resistant TB, extensively drug-resistant TB) was determined and the appropriate regimen was prescribed. Patients diagnosed with TB were started on treatment at a health facility nearest to their residence. All private health care providers received an incentive of $10 for sputum collection and transportation of each presumptive TB patient (or $5 if patients were identified and referred) and $10 per TB case detected. Diagnosis and treatment of TB were provided only at the public health facilities, free of cost to the patients.

#### 2.3.4. Recording and Reporting of the Data

At each private health facility, two registers were maintained: (i) TB symptom screening register and (ii) Sputum collection register. These registers were maintained by the nurse, who received an incentive of $20 per month. Field specialists (*n* = 7) were hired by the project to coordinate with the private clinics and visit the clinics once every month. They provided on-the-job training to private providers if needed, conducted data validation, compiled monthly reports and provided supportive supervision and monitoring. The monthly reports included aggregate numbers of people screened for symptoms, numbers with presumptive TB, numbers tested for TB using sputum microscopy and Xpert MTB/RIF and test results—all of these were disaggregated by gender. Once a patient was diagnosed with TB, the details were entered into an individual patient electronic database. This was maintained by the M&E specialist of the project. A WhatsApp group was established and all the project staff and the private providers were included in this group. One dedicated person was in charge of the WhatsApp group and was responsible for addressing any queries and providing clarifications for the providers. A call center was established, test results were shared with private providers via phone and paper-based results were transported by a courier.

### 2.4. Study Population

The study population consisted of all patients who presented at private health care facilities for any reason and were screened for TB in four selected regions (Chuy, Issyk-Kul, Naryn, Talas) and the city of Bishkek from February 2021 to March 2022.

### 2.5. Operational Definitions

All the definitions in this study are shown in Table 1.

### 2.6. Data Collection, Variables and Sources of Data

Data variables included aggregate numbers of patients screened, numbers identified as presumptive TB and numbers tested for TB, diagnosed and treated for TB. These were extracted from the monthly reports submitted by the private health facilities. For all diagnosed TB patients, we extracted patient-wise data on socio-demographic, clinical and risk factors from the electronic patient database managed by National Phthisiology Centre.

### 2.7. Data Entry and Analysis

The aggregate data were summarized as frequencies and percentages and the care cascade was presented as a flowchart. Individual patient-wise data downloaded in MS Excel were cleaned and exported to EpiData Analysis (version 2.2.2.187, EpiData Association, Odense, Denmark) for further analysis. Continuous variables were summarized using means or medians (along with standard deviation/interquartile range) as appropriate. Categorical variables were summarized using frequencies (and percentages). To determine the demographic and clinical factors associated with pre-treatment loss to follow-up and unsuccessful treatment outcomes, we conducted chi-square tests and calculated risk ratios and 95% confidence intervals as measures of association. We also calculated the delays in testing, diagnosis and treatment and expressed as median (interquartile range). A *p* value < 0.05 was considered statistically significant. Since the overall numbers and the key events of interest (for example, unsuccessful outcomes and pre-treatment loss to follow-up) were small across several sub-groups, we considered it prudent not to conduct a multivariable analysis.

## 3. Results

### 3.1. Cascade of Care

**Figure 2:**
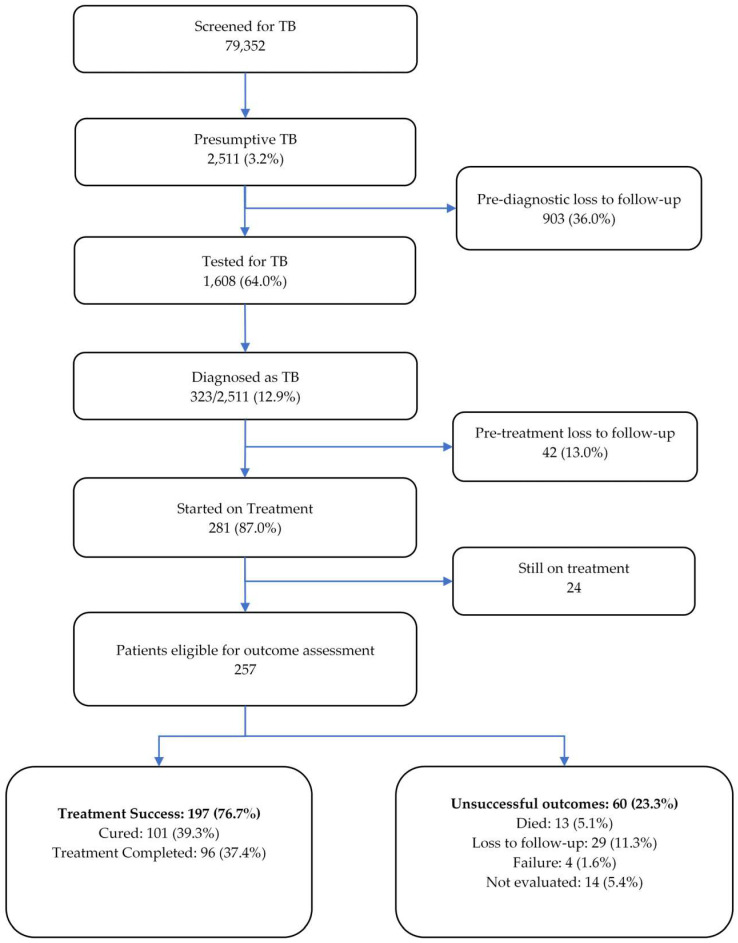
Cascade of tuberculosis care among patients screened for TB in the private health facilities in four regions and Bishkek city of Kyrgyzstan between February 2021 and March 2022.

The cascade of care is depicted in Figure 2. A total of 79,352 patients were screened for TB during the study period and among them, 2511 (3.2%) had presumptive TB. Of the latter, 1608 (64%) were tested for TB using sputum microscopy and/or Xpert MTB/RIF assay. The remaining 903 (36%) presumptive TB patients who were not tested for TB were considered as ‘pre-diagnostic loss to follow-up’. A total of 323 (12.9%) patients were diagnosed as TB and among them, 281 (87%) were started on treatment. The remaining 42 (13%) were considered ‘pre-treatment loss to follow-up’.

At the time of data collection, 24 patients were still on treatment. Among 257 patients eligible for outcome assessment, 197 patients (76.7%) had treatment success (39.3% cured and 37.4% completed treatment). Out of 60 patients (23.3%) with unsuccessful outcomes, 29 (11%) were lost to follow-up, 14 (5%) were not evaluated, 13 (5.1%) died and 4 patients (1.6%) had treatment failure.

### 3.2. Demographic, Risk and Clinical Characteristics of TB Patients

Demographic characteristics and risk factor profile of TB patients are shown in Table 2.

About 54% of patients were males and the mean (SD) age of the patients was 35 (19) years. The majority (69%) of study participants lived in Bishkek city. Regarding risk factors, 31% were unemployed, 14% had a history of contact with TB, 9% were internal migrants, 8% were smokers, 6% were external migrants, 5% had known diabetes, 4% used alcohol, 1.2% were homeless and 0.6% were ex-prisoners. Four patients were HIV-positive and among them and three (75%) were started on ART. The clinical characteristics of TB patients are presented in Table 3.

Most (87%) patients had pulmonary TB and nearly 91% were new cases. About 61% of patients were bacteriologically confirmed. The majority (72%) of patients had drug-sensitive TB, followed by MDR-TB (20%) and poly-resistant TB (7%). One patient had XDR-TB.

### 3.3. Delays in Testing, Diagnosis and Initiation of Treatment

Table 4 depicts the delays involved at different stages of the cascade of care. The proportion of patients with valid dates for the different stages ranged between 65% and 96%. The median duration between screening and registration of TB among those with valid dates was 7 days.

### 3.4. Factors Associated with Pre-Treatment Loss to Follow-Up

Factors associated with pre-treatment loss to follow-up among diagnosed TB patients are shown in Table 5. The only statistically significant variables associated with pre-treatment loss to follow-up were clinically diagnosed TB (RR = 2.05; 95% CI: 1.16–3.63) and drug sensitive TB (RR = 4.84; 95% CI: 1.53–15.28). None of the other socio-demographic characteristics or risk factors were statistically significant.

### 3.5. Factors Associated with Unsuccessful Treatment Outcomes

Given the small numbers of drug-resistant TB patients, we analyzed factors associated with unsuccessful treatment outcomes only among drug-sensitive TB patients and these are presented in Table 6. None of the variables showed a statistically significant association.

## 4. Discussion

This is the first study from Kyrgyzstan describing the cascade of care of tuberculosis patients identified in the private health sector. This adds to limited global evidence on the TB care cascade from the private sector. Traditionally, the private health sector has not been involved in TB care in The Kyrgyz Republic. One of the big achievements of this project has been to garner political commitment to create a legal and regulatory framework for engaging the private health sector. There were three key findings and we discuss them below.

First, pre-diagnostic LFU was observed in 36% of the presumptive TB patients. This is relatively high, when compared to findings from other settings. A study from South Africa reported a pre-diagnostic LFU of 18%, although it is to be noted that this study was conducted in government health facilities [9,10]. Another study from Zimbabwe reported an overall pre-diagnostic LFU of 25%, with significantly higher LFU rates observed in patients attending private-for-profit (36%) health facilities, local self-government-run council hospitals (35%) and church-run mission hospitals (25%), compared to government health facilities (14%) run by the Ministry of Health [4]. In our study, we did not have individual patient-wise data on all presumptive patients and hence were unable to assess the factors associated with pre-diagnostic LFU. The Zimbabwe study reported that pre-diagnostic LFU was higher among HIV-infected presumptive TB patients and those residing in rural areas (as compared to urban areas), reflecting poorer access to diagnostic health facilities and tools and inefficient specimen collection and transport mechanisms.

Second, pre-treatment LFU was observed in 13% of patients. Pre-treatment LFU is relatively well studied compared to pre-diagnostic LFU. Studies across the globe report that pre-treatment LFU among TB patients varied from 4% to 38% and was more common in settings from Africa (18%, 95% CI: 13–22) compared to Asia (13%, 95% CI: 10–15) [3]. Most of these studies were conducted in the public health sector. There is very limited evidence from the private-for-profit health sector on this issue, barring a study from Pakistan which reported an alarming pre-treatment LFU of 64% among smear-positive tuberculosis patients [11]. We found that pre-treatment LFU was significantly lower in drug-resistant TB patients compared to drug-sensitive TB patients, possibly reflecting higher priority accorded to drug-resistant TB patients by the NTP and better tracking and follow-up. We also found that pre-treatment LFU was higher in clinically diagnosed TB patients compared to bacteriologically confirmed TB patients. While we did not study the reasons for pre-treatment LFU, we speculate that this might indicate low risk perception among the clinically diagnosed TB patients, especially those with minimal or no symptoms and non-specific, TB-suggestive shadows on chest radiography without any bacteriological confirmation. Traditionally, such patients have received lower priority from the NTPs due to the limited role they play in community transmission. This needs further investigation.

Third, treatment outcomes were poor and nearly one-fourth of the patients started on treatment had an unsuccessful outcome. This is relatively high compared to the treatment outcomes of patients identified, diagnosed and treated in the public health sector of The Kyrgyz Republic. LFU accounted for about half of all unsuccessful outcomes, followed by ‘not evaluated’ which accounted for another 25% of unsuccessful outcomes—both indicate suboptimal mechanisms for patient tracking and follow-up and poor supervision and monitoring. We did not find any demographic and clinical factors associated with unsuccessful outcomes.

Our study had several strengths. First, this study addressed a national research priority. Second, we included all the patients cared for as part of the project without any sampling and covered four regions and the city of Bishkek city (with a large private sector). Thus, the findings reflect the ground realities. Third, we conducted and reported the study in line with STROBE (strengthening the reporting of observational studies in epidemiology) guidelines [12].

We had some limitations too. There were missing data and inconsistencies, especially with regards to variables such as dates of screening, diagnosis and treatment. The data on risk factors were self-reported by the patients. Given the lack of standard definitions of the risk factors (such as alcohol use and smoking) in the national guidelines, ‘misclassification’ of exposure status cannot be ruled out. This may partly explain the lack of associations of risk factors with unsuccessful outcomes in our analyses. The other reason for lack of statistically significant associations was low sample size. Many exposure subgroups had zero outcome events, which precluded us from conducting detailed and robust multivariable analyses. We defined pre-diagnostic LFU based on testing with sputum microscopy and or Xpert MTB/RIF assay. It is possible that many presumptive TB patients would have undergone testing using other tests (such as radiography, histopathology, ultrasound and so on) and we did not have any information on these—this might have marginally overestimated the rates of pre-diagnostic LFU.

Despite these limitations, there were many policy and practice implications. First, donor-funded private sector engagement projects, such as the one described in this study, are challenging to sustain in the long term. Given the crucial role of private sector engagement in the efforts to end TB, the NTP needs to take ownership of this initiative by including it in the national strategic plan with dedicated budgets, activities and plans to monitor the progress. Currently, there is no field in the electronic database to capture if the diagnosed patient was referred from the private sector or not. Having such a field will enable calculation of an indicator, “proportion of TB patients referred by the private sector”, on an ongoing basis. This is crucial for monitoring the engagement of private providers in TB care going forward.

Second, the recording system needs to be strengthened. The definitions of risk factors need to be standardized and communicated to the health providers involved in the care and documentation of TB patients. Measures to monitor the patient database for missing data and validation need to be put in place.

Third, qualitative research needs to be undertaken to understand the root causes of LFU at every stage (pre-diagnostic, pre-treatment and during treatment), from the perspective of both patients and providers. The results should guide future programmatic interventions. Possible reasons for pre-diagnostic and pre-treatment LFU reported in the published literature include (i) deficiencies in documentation (of addresses and phone numbers of patients) in the presumptive TB and laboratory registers, (ii) poor sputum collection and transport mechanisms, (iii) poor access to diagnostic tools and facilities, (iv) lack of awareness among patients about their TB diagnosis, (v) a lack of risk perception about the consequences of not treating TB, (vi) death in severely ill patients before starting treatment, (vii) long distances from the health facility, (viii) transport costs and (ix) dissatisfaction with the health services [13,14,15,16,17,18]. A simple programmatic intervention could be to ensure recording the addresses and phone numbers of the patients in the registers and using them to track and follow-up.

## 5. Conclusions

In conclusion, we found that there were many gaps in the cascade of care of presumptive TB patients identified and referred by the private healthcare providers in The Kyrgyz Republic. There were losses at each step of the cascade which included pre-diagnostic LFU, pre-treatment LFU and LFU during the treatment. Further qualitative enquiry is urgently needed to understand the reasons for these gaps and instituting corrective measures.

## Figures and Tables

**Table 1:** Operational definitions.

Term	Definition
Presumptive TB	People with cough ≥ 2 weeks People with chest X-ray shadows suggestive of TB People with other symptoms (fever, chest pain, weight loss, night sweats) suggestive of TB and deemed to be ‘presumptive TB’ based on the clinical discretion of the physician
Tested for TB	Presumptive TB tested using Xpert MTB/RIF and/or sputum microscopy
Diagnosis of TB	Patient positive for acid-fast bacilli on microscopy Patients positive for Mycobacterium Tuberculosis (MTB) on Xpert MTB/RIF Patients negative for MTB on microscopy and Xpert MTB/RIF, but clinically diagnosed with TB based on chest radiography findings and clinical assessment Patient diagnosed as extra pulmonary TB based on clinical assessment and other relevant investigations (histology, radiography)
Rifampicin-Resistant TB (RR-TB)	Patients with rifampicin resistance result on Xpert MTB/RIF
Treatment for TB	Diagnosed TB started on a TB treatment regimen (either with first-line drugs or second-line drugs)
Successful outcomes	Cured Treatment completed
Unsuccessful outcomes	Loss to follow-up Died Treatment Failure Not evaluated

**Table 2:** Demographic characteristics and risk factor profile of tuberculosis patients diagnosed from those screened and referred by the private health facilities in four selected regions and Bishkek city of Kyrgyzstan between February 2021 and March 2022 (N = 323).

Characteristics	N	(%)
Age		
0–14	44	(13.6)
15–34	136	(42.1)
35–64	107	(33.1)
65 and above	36	(11.1)
Gender		
Male	174	(53.9)
Female	149	(46.1)
Region		
Bishkek	224	(69.3)
Chuy	77	(23.8)
Talas	15	(4.6)
Naryn	6	(1.9)
Issyk-Kul	1	(0.3)
Risk factors *		
Contact	45	(13.9)
Smoking	27	(8.4)
Alcohol use	13	(4)
Drug use	0	(0)
Homeless	4	(1.2)
Unemployed	99	(30.7)
Ex-Prisoner	2	(0.6)
Diabetes	15	(4.6)
External migrant	19	(5.9)
Internal migrant	30	(9.3)
HIV **-positive	4	(1.2)
Receipt of ART **	3	(75) **

* A patient can have more than one risk factor; ** HIV = Human Immunodeficiency Virus; ART = antiretroviral therapy (percentage calculated among HIV-positive).

**Table 3:** Clinical characteristics of tuberculosis patients diagnosed from those screened and referred by the private health facilities in four selected regions and Bishkek city of Kyrgyzstan between February 2021 and March 2022 (N = 323).

Characteristics	N	(%)
Type of TB		
Pulmonary	280	(86.7)
Extra pulmonary	43	(13.3)
Registration category		
New	295	(91.3)
Relapse	15	(4.6)
Treatment after LTFU *	1	(0.3)
Treatment after Failure	2	(0.6)
Others	7	(2.3)
Not recorded	3	(0.9)
Clinical classification		
Bacteriologically confirmed	196	(60.7)
Clinically diagnosed	127	(39.3)
Drug resistance		
MDR-TB	64	(19.8)
XDR-TB	1	(0.3)
Poly resistant TB	24	(7.4)
Drug-sensitive TB *	233	(72.1)
Not recorded	1	(0.3)

MDR-TB = multidrug-resistant TB; XDR-TB = extensively drug-resistant TB; * This includes people bacteriologically confirmed to have drug-sensitive TB and those clinically diagnosed and presumed to be having drug-sensitive TB and treated with first-line drugs.

**Table 4:** Delays in testing, diagnosis and treatment initiation among tuberculosis patients diagnosed from those screened and referred by private health facilities in four selected regions and Bishkek city of Kyrgyzstan between February 2021 and March 2022 (N = 323).

Duration (Days)	Number Eligible	Number (%) with Valid Dates	Median (IQR)
Screening to sputum collection	323	258 (80)	1 (0–6)
Sputum collection to diagnosis	323	211 (65)	3 (1–8)
Diagnosis to start of treatment	281	203 (72)	0 (0–1)
Start of treatment to registration	281	241 (86)	0 (0–3)
Date of screening to registration	281	269 (96)	7 (3–19)

**Table 5:** Factors associated with pre-treatment loss to follow-up among diagnosed tuberculosis patients diagnosed from those screened and referred by the private health facilities in four selected regions and Bishkek city of Kyrgyzstan between February 2021 and March 2022 (N = 323).

Variables	Total	PTLFU *		Crude RR	95% CI
		N	(%)		
Total	323	42	(13.0)		
Age group (years)					
0–14	44	7	(15.9)	2.86	0.63–12.94
15–34	136	22	(16.2)	2.91	0.71–11.80
35–64	107	11	(10.3)	1.85	0.43–7.95
65 and above	36	2	(5.6)	Ref	
Sex					
Male	174	28	(16.1)	1.71	0.94–3.13
Female	149	14	(9.4)	Ref	
Region					
Bishkek	224	32	(14.3)	1.41	0.72–2.76
Others	99	10	(10.1)	Ref	
Contact					
No	278	39	(14.0)	2.10	0.67–6.52
Yes	45	3	(6.7)	Ref	
Smoking					
No	296	42	(14.2)	NA	NA
Yes	27	0	(0)		
Alcohol use					
No	310	42	(13.5)	NA	NA
Yes	13	0	(0)		
Drug use					
No	323	42	(13)	NA	NA
Yes	0	0	(0)		
Homeless					
No	319	42	(13.6)	NA	NA
Yes	4	0	(0)		
Unemployed					
No	224	42	(18.8)	NA	NA
Yes	99	0	(0)		
Ex-prisoner					
No	321	42	(13.1)	NA	NA
Yes	2	0	(0)		
Diabetes					
No	308	42	(13.6)	NA	NA
Yes	15	0	(0)		
External Migration					
No	304	38	(12.5)	Ref	
Yes	19	4	(21.1)	1.68	0.67–4.23
Internal Migration					
No	293	42	(14.3)	NA	NA
Yes	30	0	(0)		
HIV status					
No	319	42	(13.2)	NA	NA
Yes	4	0	(0)		
Type of TB					
Pulmonary	280	38	(13.6)	1.45	0.54–3.88
Extra pulmonary	43	4	(9.3)	Ref	
Registration category					
New	295	37	(12.5)	1.50	0.38–5.86
Previously treated	24	2	(8.3)	Ref	
Clinical classification					
Bacteriologically confirmed	196	18	(9.2)	Ref	
Clinically diagnosed	127	24	(18.9)	2.05	1.16–3.63
Drug resistance					
DR-TB	89	3	(3.4)	Ref	
DS-TB	233	38	(16.3)	4.84	1.53–15.28

* PTLFU = Pre-treatment loss to follow up; HIV = Human Immunodeficiency Virus; DR-TB = drug-resistant tuberculosis and includes multidrug-resistant TB, extensively drug-resistant TB and poly-resistant TB; DSTB = drug sensitive TB; NA = not applicable; risk ratios in bold are for statistically significant associations (*p* < 0.05); RR = risk ratio; CI = confidence intervals.

**Table 6:** Factors associated with unsuccessful treatment outcomes among drug-sensitive TB patients diagnosed and started on treatment among those screened and referred by the private health facilities in four selected regions and Bishkek city of Kyrgyzstan between February 2021 and March 2022 (N = 195).

Factors	Total	Unsuccessful Outcomes *		Crude RR	95% CI
		N	(%)		
Total	195	46	(23.6)		
Age group (years)					
0–14	24	3	(12.5)	Ref	
15–34	79	15	(19.0)	1.51	0.47–4.80
35–64	66	20	(30.3)	2.42	0.79–7.42
65 and above	26	8	(30.8)	2.46	0.73–8.21
Sex					
Male	103	26	(25.2)	1.16	0.69–1.93
Female	92	20	(21.7)	Ref	
Region					
Bishkek	139	34	(24.5)	Ref	
Others	56	12	(21.4)	0.88	0.49–1.57
Contact					
No	168	41	(24.4)	1.31	0.57–3.03
Yes	27	5	(18.5)	Ref	
Smoking					
No	180	45	(25.0)	3.75	0.55–25.33
Yes	15	1	(6.7)	Ref	
Alcohol use					
No	186	42	(22.6)	Ref	
Yes	9	4	(44.4)	1.96	0.9–4.28
Drug use					
No	195	46	(23.6)	NA	NA
Yes					
Homeless					
No	192	45	(23.4)	Ref	
Yes	3	1	(33.3)	1.42	0.28–7.19
Unemployed					
No	132	33	(25.0)	Ref	
Yes	63	13	(20.6)	0.83	0.47–1.46
Ex-prisoner					
No	194	46	(23.7)	NA	NA
Yes	1	0	(0)		
Diabetes					
No	185	43	(23.2)	Ref	
Yes	10	3	(30.0)	1.29	0.48–3.44
External Migration					
No	183	43	(23.5)	Ref	
Yes	12	3	(25.0)	1.06	0.38–2.93
Internal Migration					
No	174	44	(25.3)	Ref	
Yes	21	2	(9.5)	0.37	0.09–1.44
HIV status					
No	193	46	(23.8)	NA	NA
Yes	2	0	(0)		
Type of TB					
Pulmonary	167	42	(25.1)	1.76	0.68–4.52
Extra pulmonary	28	4	(14.3)	Ref	
Registration category					
New	181	41	(22.7)	Ref	
Previously treated	14	5	(35.7)	1.57	0.74–3.34
Clinical classification					
Bacteriologically confirmed	106	30	(28.3)	1.57	0.91–2.69
Clinically diagnosed	89	16	(18.0)	Ref	

* Unsuccessful outcomes = these include death, treatment failure, loss to follow-up and not evaluated; HIV = Human Immunodeficiency Virus; DR-TB = drug-resistant tuberculosis and includes multidrug-resistant TB, extensively drug-resistant TB and poly resistant TB; DSTB = drug sensitive TB; NA = not applicable; RR = risk ratio; CI = confidence intervals.

## Data Availability

Data available on request from the corresponding author of the study.
